# : A case report of re-challenge of immune checkpoint inhibitors after immune-related neurological adverse events: Review of literature

**DOI:** 10.1097/MD.0000000000030236

**Published:** 2022-09-09

**Authors:** Heesung Moon, Seul-Gi Kim, Seung Ki Kim, Jinkwon Kim, Seung Ryeol Lee, Yong Wha Moon

**Affiliations:** a CHA University, School of Medicine, Seongnam, Korea; b Hematology and Oncology, Department of Internal Medicine, CHA Bundang Medical Center, CHA University, Seongnam, Korea; c Department of Surgery, CHA Bundang Medical Center, CHA University, Seongnam, Korea; d Department of Neurology, Yongin Severance Hospital, Yonsei University College of Medicine, Seoul, Korea; e Department of Urology, CHA Bundang Medical Center, CHA University, Seongnam, Korea.

**Keywords:** adverse drug events, cancers, immunotherapy, case report, nervous system toxicity, neurologic manifestations

## Abstract

The indications for immune checkpoint inhibitors (ICIs) are expanding for various cancers because of their durable responses and tolerable safety profiles. Immune-related adverse events (irAEs), including neurological adverse events (nAEs), are associated with ICIs therapy. However, there have been few studies on whether re-challenge with ICIs can be clinically acceptable after neurological AE has improved.

**Patient concerns:**

A 69-year-old woman with recurrent ovarian cancer undergoing palliative chemotherapy was admitted to our hospital with sudden development of diplopia, dizziness, and gait instability. The patient was administered ICI therapy with anti-angiogenic agents for 9 weeks for 3 cycles.

**Diagnosis:**

We performed neurological examination, brain imaging, nerve conduction studies, and serology tests. The patient was diagnosed with Guillain–Barré syndrome variant, an immune-mediated polyneuropathy characterized by a triad of ataxia, areflexia, and ophthalmoplegia.

**Intervention:**

After prompt discontinuation of pembrolizumab, the patient was taken intravenous methylprednisolone (2 mg/kg) was administered for 5 days, and her symptoms were partially resolved. With the addition of immunoglobulin 0.4 g/kg for 5 days, her symptoms gradually improved.

**Outcomes:**

The patient’s neurological symptoms improved after immunosuppressive therapy, without sequelae. The NCV showed normal nerve conduction. Unfortunately, because there was little evidence for pembrolizumab rechallenge, pembrolizumab therapy was permanently discontinued, and the tumors eventually progressed.

## 1. Introduction

Immune checkpoint inhibitors (ICIs), including antiprogrammed cell death-1/ligand-1 (PD-1/PD-L1) and anti-cytotoxic T lymphocyte antigen-4 (CTLA-4), block the pathways that downregulate the activity of T lymphocytes. In recent years, PD-1/PD-L1 inhibitors such as pembrolizumab, nivolumab, atezolizumab, durvalumab, and avelumab, and CTLA-4 inhibitors such as ipilimumab have been approved by the FDA for the treatment of various cancers.^[1,2]^ In particular, PD-L1-expression (≥1%) patients with advanced non-small cell lung cancer (NSCLC) who were treated with pembrolizumab as the first-line treatment showed a 5-year overall survival rate of approximately 23%, compared to a historical control of ~5% (per SEER 2008–2014), before the introduction of anti–PD-1 therapy in the KEYNOTE-001 study.^[3]^ ICIs have been approved for the treatment of various cancer types and used for the long term, as shown in KEYNOTE-001, owing to their durable response and tolerable safety profiles. Although ICIs generally show acceptable toxicities,^[4]^ they sometimes induce immune-related adverse events (irAEs), including skin disorders, endocrinopathy, colitis, hepatitis, nephritis, pneumonitis, and neurological adverse events (nAEs).^[5]^ Although rare, in the expanding era of ICIs, nAEs might become more common. There have been few studies on the clinical course of nAE, and the re-challenge of ICIs after nAE has improved. Most medical oncologists are interested in whether ICIs can be re-administered after neurological AEs have improved.

We reviewed the nAE cases, including our case of Guillain–Barré syndrome variant, and the evidence for re-challenge of the ICI by searching keywords in PubMed.

## 2. Case report: Guillain–Barré syndrome variant

### 2.1. Patient concerns

In March 2017, a 69-year-old woman with refractory metastatic epithelial ovarian cancer was treated with pembrolizumab 200 mg in combination with bevacizumab 15 mg/kg after failure of many lines of systemic chemotherapy, with the rationale of potential synergism of ICIs and angiogenesis inhibitors.^[6]^ The prior anticancer treatment history comprised neoadjuvant paclitaxel and carboplatin, followed by debulking surgery and adjuvant chemotherapy between February and July 2011. From June 2013, she received palliative paclitaxel and carboplatin as front-line therapy for a platinum-sensitive recurrent disease. Subsequently, liposomal doxorubicin, topotecan, gemcitabine, and vinorelbine were administered as subsequent lines of therapy between February 2016 and February 2017 for platinum-resistant ovarian cancer.

**Figure 1.**
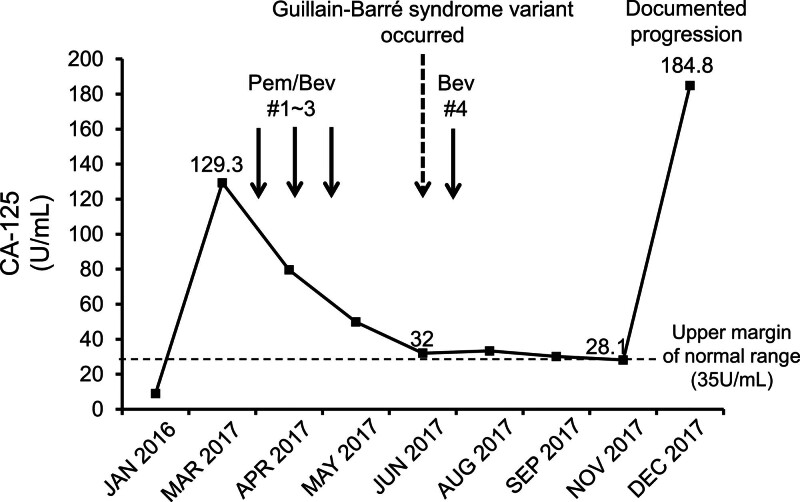
Dynamics of serum CA-125 level by the time showed a decrease in CA-125 after treatment despite the discontinuation of pembrolizumab after the neurologic adverse event. # = cycle, Pem = pembrolizumab, Bev = bevacizumab.

After 3 cycles of pembrolizumab and bevacizumab, which was 9 weeks from the start, a partial response (41% reduction of tumor burden) with decreased CA125 (Fig. 1) was achieved. However, the patient was admitted to the hospital in June 2017 with sudden development of diplopia, dizziness, and gait instability. Neurological examination revealed ophthalmoparesis of the superior and inferior recti as well as the lateral recti of both eyes. Both pupils were round, equal, and promptly reactive to the light. Except for ophthalmoparesis, all other cranial nerve functions were intact, and the facial muscles were symmetrical. She had no headache or ocular pain and denied a history of diarrhea, infectious events, or head trauma. Although we did not observe focal motor deficits in the limbs, the patient complained of severe gait instability. Decreased deep tendon reflexes and impaired proprioception were observed in both lower limbs.

### 2.2. Diagnosis

We found no abnormal findings on MRI and MR angiography of the brain. There were no abnormal features in the neostigmine test and repetitive nerve stimulation test for the differential diagnosis of myasthenia gravis, there was no abnormal feature. A nerve conduction study found a pattern of sensorimotor polyneuropathy with predominant lower-limb involvement. Despite the normal nerve conduction velocity (NCV) in the left median and ulnar motor nerves, low sensory nerve action potentials, and slow NCV were observed in the left median and ulnar sensory nerves. An important observation was the unobtainable motor NCV on the bilateral peroneal motor nerves with prolonged terminal latency, low compound muscle action potential, and slow NCV on the bilateral tibial motor nerves. Low sensory nerve action potentials and slow NCV on both the superficial peroneal sensory and left sural sensory nerves were assessed. There was no sensory nerve action potential in the right sural sensory nerve. A nerve conduction test on the right upper limb was not applicable because of the right arm fracture history. The ganglioside IgM panel test results were as follows: anti-GD1b IgM antibody was positive, whereas anti-GQ1b IgM was negative. Taken together, the patient’s clinical features resembled the Guillain–Barré syndrome variant, an immune-mediated polyneuropathy characterized by a triad of ataxia, areflexia, and ophthalmoplegia.

### 2.3. Interventions

We started intravenous methylprednisolone (2 mg/kg) for 5 days, and her symptoms were partially resolved. With the addition of immunoglobulin 0.4 g/kg for 5 days, her symptoms gradually improved. The patient received an additional cycle of bevacizumab monotherapy. There was little evidence for rechallenge with ICIs, and pembrolizumab was permanently discontinued.

### 2.4. Outcomes

The patient recovered from most neurological symptoms, such as diplopia, dizziness, and gait disturbance, after immunosuppressive therapy. No long-term morbidities or neurological sequelae were observed. The NCV showed normal nerve conduction.

Despite 6 months of pembrolizumab treatment, she showed 10 months of progression-free status until December 2017, when the CA125 level started to increase and peritoneal seeding metastases had progressed.

### 2.5. Review of the literature

We have reviewed the literature of 35 case reports on PubMed after searching keywords: “adverse immune event anti-PD-1, anti-PD-L1 or anti-CTLA-4,” “neurological adverse immune event anti-PD-1, anti-PD-L1 or anti-CTLA-4,” “adverse immune event pembrolizumab, nivolumab, atezolizumab, avelumab, durvalumab, tremelimumab or ipilimumab” and “neurological adverse immune event pembrolizumab, nivolumab, atezolizumab, avelumab, durvalumab, tremelimumab or ipilimumab” For further analyses, we included 15 out of 35 cases with nAEs with our case of Guillain–Barré syndrome variant (Table 1). Twenty case reports were excluded due to insufficient data on the neurological course and clinical association with ICIs.

**Table 1:** Description of neurological adverse events in consecutive cases (*N* = 15).

Refs	Patient	Agents/Dose/Cycle	nAE	Symptom progression with treatment	Tumor response (Response duration)
^[7]^	78 yr old male, lung squamous cell carcinoma	Nivolumab, 3 mg/kg, 14 cycle (discontinued permanently)	Autoimmune encephalitis	Complete recovery with methylprednisolone	PR (9 mo)
^[8]^	50 yr old female, melanoma	Nivolumab, 1 mg/kg, + Ipilimumab, 3 mg/kg, 1 cycle (discontinued permanently)	Autoimmune encephalitis	Complete recovery with methylprednisolone 1000 mg i.v., 0.4mg/kg/day IVIG (no I mprovement) and rituximab 1000 mg i.v.	PR (4 mo)
^[8]^	65 yr old male, small cell lung cancer	Nivolumab, 1 mg/kg + Ipilimumab, 3 mg/kg, 1 cycle (discontinued permanently)	Autoimmune limbic encephalitis	Complete recovery with prednisolone 60 mg/day	PR (2 mo)
^[9]^	69 yr old female, melanoma	Pembrolizumab, 2 mg/kg, 3 cycles (discontinued permanently)	Paralysis, Myasthenia Gravis	Not improved with methylprednisolone 1000mg i.v., and plasmapheresis; death at 4 month	PR
^[9]^	38 yr old male, melanoma	Pembrolizumab, 2 mg/kg, 35 wk (paused for 3 wk)*	Polyradiculitis	Partially resolved with prednisolone 1g i.v.	PD
^[10]^	58 yr old male, lung adenocarcinoma	Nivolumab, 150 mg/body, 4wks (discontinued permanently)	Akathisia	Not improved with methylprednisolone pulse therapy; death	SD
^[11]^	78 yr old male, melanoma	Ipilimumab, 3 mg/kg, 4 cycles (discontinued)	Polyradiculoneuropathy	Partially resolved with methyprednisolone 1mg/kg	PD (3 mo)
^[11]^	79 yr old female, melanoma	Nivolumab, 3 mg/kg, 5 cycles (discontinued)	Phrenic neuropathy	Complete recovery with IVIG for 4month	CR (7 mo)
^[11]^	67 yr old male, melanoma	Ipilimumab, 3 mg/kg + Nivolumab,1 mg/kg,1 cycle (discontinued)	Autoimmune bilateral lumbar plexopathy	Complete recovery with methylprednisolone 2mg/kg for 2 month	PR (2 mo)
^[12]^	51 yr old male, melanoma	Ipilimumab, 1 cycle (discontinued)	Immune-related meningitis	Complete recovery with dexamethasone oral 8mg/day,	PD
^[13]^	57 yr old female, melanoma	Ipilimumab, 3 mg/kg, 10wks (discontinued)	Guillain- Barre Syndrome	Complete recovery with methylprednisolone i.v. 80mg/day	PR
^[14]^	69 yr old female, melanoma	Ipilimumab, 3 mg/kg, completed 4 cycles (continued)*	Myasthenia Gravis	Partially resolved with methylprednisolone i.v.2mg/kg and plasmapheresis	PR
^[15]^	81 yr old male, melanoma	Pembrolizumab, 2 mg/kg, 8 cycles (continued, temporary discontinuedat4th)*	Myasthenia Gravis, ocular	Complete recovery with prednisolone 25mg/day	CR
^[15]^	86 yr old female, melanoma	pembrolizumab, 2 mg/kg, 3 cycles (discontinued permanently)	Myasthenia Gravis, ocular	Resolved with methylprednisolone i.v. 500mg/day, followed by oral prednisolone	CR (3 mo after postdiscontinuation)
Our case	69 yr old female, ovarian cancer	Pembrolizumab 2 mg/kg, 3 cycles (discontinued)	Guillain- Barre Syndrome, variant	Partially resolved with methylprednisolone 125mg i.v., IVIG 0.4mg/kg	PR (7 mo)

* The immune checkpoint inhibitor therapy was continued with or without pause when the patients had ongoing neurologic adverse events in these 3 cases.

Refs = references, nAE = neurological adverse events, IVIG = intravenous immunoglobulin, CR = complete response, PR = partial response, SD = stable disease, PD = Progressive disease.

### 2.6. Management and disease course of neurological adverse events

Of the 15 cases, there seemed to be no specific pattern between the onset timing and ICIs treatment. The onset of the nAEs ranged from 1 cycle to 12 cycles (median of 6 weeks) with or without ICI pauses. The severity of nAEs was reported as grade 3 or 4 toxicity in half of the cases. These results should be interpreted with caution because of publication bias toward more severe toxicities being reported.

The majority of patients’ symptoms were effectively reversed: completely resolved in 9, partially resolved in 4, and not improved in 2 of the 15 cases. With corticosteroid treatment, neurologic symptoms usually improve within 24 h to a week. Although additional therapies were not listed in the US FDA labeling, intravenous immunoglobulin (IVIG) was administered in 3 cases if the patient’s neurologic symptoms did not improve despite corticosteroid treatment. After IVIG administration, 1 patient with phrenic neuropathy completely recovered.^[11]^ However, in the other cases with myasthenia gravis^[9]^ and our case, the Guillain–Barre Syndrome variants did not fully recover.

### 2.7. Tumor response

An interesting observation of these case series was that most of the patients with nAEs showed good response to the immune checkpoint inhibitor therapy: 3 complete responses, 9 partial responses, 1 stable disease, and 2 progressive diseases in a total of 15 cases.

Of the 9 cases in which response duration was mentioned, the median response duration was 4 (range, 2–9) months, even though ICIs were discontinued after neurologic toxicities developed. Examples of durable response after discontinuation of therapy were nivolumab treatment for lung squamous cell carcinoma showing substantial tumor regression after 2 months of therapy followed by 9 months of ICI.^[7]^ Another case of malignant melanoma showed a complete response at 7 months, and no new metastasis was detected 54 months after discontinuation from the first 5 cycles of nivolumab therapy.^[11]^ In addition, our case of ovarian cancer showed a partial response and 10 months of progression-free survival after discontinuation of the first 3 cycles of pembrolizumab.

### 2.8. Re-challenge with immune checkpoint inhibitors

In 12 of the 15 case series, ICI therapy was immediately stopped and permanently discontinued when patients showed neurological toxicities, whereas, in the remaining 3 cases, ICI was either paused temporarily or continued despite the ongoing neurological symptoms. In one case, irAEs presented 35 weeks after the initiation of pembrolizumab treatment for malignant melanoma.^[9]^ The patient’s condition improved after prednisolone therapy. Pembrolizumab therapy was continued after 3 weeks of steroid treatment without any aggravation of neurological symptoms. In another case of melanoma, pembrolizumab was temporarily discontinued after the 4th cycle and then resumed up to 8th cycle after the patient had completely recovered from neurologic symptoms.^[15]^ During retreatment with pembrolizumab in this case, the neurological symptoms did not relapse. In another case, ipilimumab was continued even after neurological toxicity was initially observed after the 2nd cycle. Ipilimumab treatment was continued up to 4th cycles.^[14]^ Neurological symptoms were controlled after partial recovery with methylprednisolone and plasmapheresis.

Recently, a retrospective study investigating the safety of re-challenge of ICIs after irAEs (*N* = 40), not specifically for nAEs, the recurrence rate of irAEs was approximately 55%, and most symptoms were not more severe than the initial occurrence.^[16]^ A shorter time to the initial irAE after ICI treatment was associated with a higher rate of occurrence of a second irAE (9 vs. 15 weeks; *P* = 0.04). Although the authors concluded that the risk-benefit ratio for ICI re-challenge may be acceptable, it cannot be simply applicable to clinics in cases of nAE because nAEs might be more morbid than other irAEs. Another case series of re-challenge after irAEs were listed in Table 2. The retrospective and heterogeneous nature of these studies is a potential caveat. None of the guidelines dealt with re-challenge, especially for moderate to severe nAEs. Current evidence cannot justify the routine re-administration of ICIs. Nevertheless, re-challenge with ICIs after nAEs needs further investigation to prevent any loss of opportunity for treatments.

**Table 2:** Case series of re-challenge after the immune-related advised events.

Refs	Total Number	Agents/median duration of discontinuation	Incidence of non-recurring irAE	Recurrence rate of same irAE/Occurrence rate of new irAE	Grade 3 or higher irAE after re-challenge	Turmor response at re-challenge (Number of patients of CR/PR/SD/PD)
^[16]^	40	Anti-PD-1 or Anti PD-L1/ 3.8wks	18 (45%)	17 (42.5%)/ 5(12.5%)	3 (14%)	(0/13/15/9)
^[17]^	80	Anti-PD-1/ 8.2 wks	40 (50%)	14 (18%)/ 36 (32%)	14 (18%)	(CR+PR 56/15/9)
^[18]^	10	Anti-PD-1 or Anti-CTLA-4/ 24 wks	4 (40%)	Total 6 (60%)*	unknown	unknown
^[19]^	67	Anti-PD-1/ unknown	44 (66%)	2 (3%)/ 23 (34%)	14 (21%)	unknown
^[20]^	2	Anti-PD-1 or Anti-PD-L1/ 30 wks	2 (100%)	Total0 (0%)*	0 (0%)	(0/1/0/1)

* It was difficult to distinguish whether it was the same irAE or a new irAE.

Refs = references, nAE = neurological adverse events, IVIG = intravenous immunoglobulin, CR = complete response, PR = partial response, SD = stable disease, PD = Progressive disease.

### Author contributions

Seul-Gi Kim: Conceptualization, Investigation, Formal analysis.

Heesung Moon: Conceptualization, Investigation, Formal analysis.

Seung Ki Kim: Investigation, Formal analysis.

Jinkwon Kim: Conceptualization, Investigation.

Seung Ryeol Lee: Conceptualization, Investigation, Formal analysis.

Yong Wha Moon: Conceptualization, Investigation, Formal analysis.
